# Functional CD1d and/or NKT cell invariant chain transcript in horse, pig, African elephant and guinea pig, but not in ruminants

**DOI:** 10.1016/j.molimm.2008.12.009

**Published:** 2009-04

**Authors:** Frank A. Looringh van Beeck, Peter Reinink, Roel Hermsen, Dirk M. Zajonc, Marielle J. Laven, Axel Fun, Milana Troskie, Nico J. Schoemaker, Darshana Morar, Johannes A. Lenstra, Lonneke Vervelde, Victor P.M.G. Rutten, Willem van Eden, Ildiko Van Rhijn

**Affiliations:** a Department of Infectious Diseases and Immunology, Faculty of Veterinary Medicine, University Utrecht, Utrecht, The Netherlands; b Division of Cell Biology, La Jolla Institute for Allergy and Immunology, La Jolla, CA, USA; c Department of Veterinary Tropical Diseases, Faculty of Veterinary Science, University of Pretoria, South Africa; d Department of Clinical Sciences of Companion Animals, University Utrecht, Utrecht, The Netherlands; e Institute of Risk Assessment Science, Utrecht, The Netherlands

**Keywords:** Other animals, T cell Receptors, Comparative immunology/evolution

## Abstract

CD1d-restricted invariant natural killer T cells (NKT cells) have been well characterized in humans and mice, but it is unknown whether they are present in other species. Here we describe the invariant TCR α chain and the full length CD1d transcript of pig and horse. Molecular modeling predicts that porcine (po) invariant TCR α chain/poCD1d/α-GalCer and equine (eq) invariant TCR α chain/eqCD1d/α-GalCer form complexes that are highly homologous to the human complex. Since a prerequisite for the presence of NKT cells is the expression of CD1d protein, we performed searches for CD1D genes and CD1d transcripts in multiple species. Previously, cattle and guinea pig have been suggested to lack CD1D genes. The CD1D genes of European taurine cattle (*Bos taurus*) are known to be pseudogenes because of disrupting mutations in the start codon and in the donor splice site of the first intron. Here we show that the same mutations are found in six other ruminants: African buffalo, sheep, bushbuck, bongo, N’Dama cattle, and roe deer. In contrast, intact CD1d transcripts were found in guinea pig, African elephant, horse, rabbit, and pig. Despite the discovery of a highly homologous NKT/CD1d system in pig and horse, our data suggest that functional CD1D and CD1d-restricted NKT cells are not universally present in mammals.

## 1 Introduction

CD1d proteins are expressed on the surface of a variety of antigen presenting cells and non-hematopoietic cells, and present cellular self-lipids and exogenous lipids with an α-anomerically linked sugar to T cells with a highly conserved, invariant TCR, NKT cells. CD1d−/− mice have no detectable mature NKT cells (Chen et al., 1997; Gapin et al., 2001), showing that functional CD1D genes are a prerequisite for their development. NKT cells have been implicated in oral tolerance, autoimmunity, dendritic cell maturation, tumor surveillance, and anti-microbial immunity. Natural exogenous ligands for NKT cells presented by CD1d have been identified, such as GSL-I from *Sphingomonas* species (Kinjo et al., 2005) and BbGL-II from *Borrelia burgdorferi* (Kinjo et al., 2006). The entire population of NKT cells can be activated strongly by the synthetic ligand α-galactosylceramide (α-GalCer) (Kawano et al., 1997), which is considered a universal super agonist for NKT cells. It has been suggested that the CD1d/NKT system evolved to cope with pathogens that produce antigens with α-glycosidic linkages (Kinjo et al., 2005), but there is only limited supportive data available.

*Sphingomonas* species contain antigens that are presented by CD1d to NKT cells. *Sphingomonas*, a genus that does not include highly pathogenic bacteria, belongs to the class of α-proteobacteria. This class of bacteria contains peptidoglycan and LPS-negative bacteria, including pathogenic tick-borne genera: *Rickettsia*, *Anaplasma*, and *Ehrlichia*, all causing morbidity and mortality in livestock. Unfortunately, none of these bacteria has been studied closely enough to determine whether they contain antigens for NKT cells. *Ehrlichia ruminantium* (formerly named *Cowdria ruminantium* (Dumler et al., 2001)), causes heartwater (cowdriosis), *Anaplasma bovis* (formerly named *Ehrlichia bovis*) causes bovine ehrlichiosis, and *A. marginale* and *A. centrale* cause bovine anaplasmosis, and these diseases are major problems in the livestock industry in sub-Saharan Africa. Some indigenous African breeds of cattle are more resistant to heartwater and anaplasmosis than other breeds, but this can be explained by higher resistance to the vector (ticks of the genus *Amblyomma*). All breeds of cattle will develop clinical disease once they get infected. Since the aforementioned bacterial pathogens do not carry the signature danger molecules LPS and peptidoglycan, recognition by the innate immune system other than TLRs, like the CD1d/NKT system, may be of crucial importance in the early defense against these pathogens.

It has been suggested that the group 1 CD1 proteins (CD1a, CD1b, CD1c) are not universally present in all species, whereas group 2 CD1 proteins (CD1d) are. CD1D genes have indeed been found in most mammalian species studied, including primates like humans and chimpanzees (*Pan troglodytes*), African green monkeys (*Chlorocebus sabaceus*) and rhesus macaques (*Macaca mulatta*) (Saito et al., 2005), mice (*Mus musculus*) (Bradbury et al., 1988), rats (*Rattus norvegicus*) (Ichimiya et al., 1994), cottontail rabbits (*Sylvilagus floridanus*) (Calabi et al., 1989), sheep (*Ovis aries*) (Rhind et al., 1999), and pigs (*Sus scrofa*) (Eguchi-Ogawa et al., 2007). However, not all of these genes have been shown to lead to functional transcripts or proteins yet. CD1 genes have also been discovered in chickens (*Gallus gallus*) (Maruoka et al., 2005; Miller et al., 2005; Salomonsen et al., 2005), but chicken CD1 genes could not be classified according to the existing isoforms, and are therefore named CD1.1 and CD1.2. There are two species that have till now been suggested to have no functional CD1D genes. Before the availability of its genome, the guinea pig (*Cavia porcellus*) family of CD1 genes had been well characterized, but a CD1D gene was not identified (Dascher et al., 1999). In cattle, two CD1D genes have been identified, named CD1D1 and CD1D2, but these are in fact pseudogenes (Van Rhijn et al., 2006). The two CD1D pseudogenes that were identified both contain a mutated start codon and an unspliceable intron. In this paper we describe CD1D pseudogenes in N’Dama cattle, and five other ruminants, including sheep, which had previously been assumed to have functional CD1D genes. Functional CD1d transcripts were identified in guinea pig, African elephant, horse, rabbit, and pig.

NKT cells can be distinguished by their highly conserved invariant TCR. The NKT cell population can be visualized by flow cytometric analysis using fluorescently labeled CD1d tetramers loaded with α-GalCer that interact with the NKT cell TCR. Human and murine CD1d tetramers are known to stain human and murine NKT cells, also in a species cross-reactive manner, so it is possible that these tetramers also recognize NKT cells in other species. However, the lack of species cross-reactive staining does not prove absence of NKT cells. Alternatively, evidence for the existence of NKT cells in a species might come from TCR α chain sequences. Recent data on the molecular interactions between α-GalCer-loaded CD1d and the invariant TCR (Borg et al., 2007; Kjer-Nielsen et al., 2006; Scott-Browne et al., 2007) have provided clear insights in these interactions and allow detailed predictions on whether CD1d and TCR protein sequence homologs that are found in other species, like dog and horse as described in this paper, are likely to be true functional homologs.

Our data provide supportive evidence that functional CD1d transcripts and/or NKT cells are present in several mammalian species, but not in ruminants. This shows that the CD1d/NKT system is not universally present as previously thought. The CD1d/NKT system may be lacking in ruminants altogether, providing a possible explanation for their high sensitivity to *Rickettsia*, *Anaplasma*, and *Ehrlichia*.

## 2 Materials and methods

### 2.1 Animals and nucleic acid samples

Genomic DNA from sheep (*Ovis aries*), African buffalo (*Syncerus caffer*), bushbuck (*Tragelaphus scriptus*), and bongo (*Tragelaphus eurycerus*), was freshly isolated from whole, heparinized blood, using the Wizard Genomic DNA Purification Kit (Promega). Genomic DNA from N’Dama cattle (*Bos taurus*) and roe deer (*Capreolus capreolus*) were isolated at least 10 years ago. DNA from N’Dama cattle was a gift of Dr. Bradley (Trinity College, Dublin, Ireland). First strand cDNA was synthesized with Multiscribe reverse transcriptase (Applied Biosystems) from RNA isolated with the Qiagen RNAEasy kit from freshly isolated PBMC from cattle, sheep, pig (*Sus scrofa*), horse (*Equus caballus*), guinea pig (*Cavia porcellus*), cat (*Felis catus*), rabbit (*Oryctolagus cuniculus*), and African elephant (*Loxodonta africana*). Animal-derived materials were obtained and collected according to the regulations of the Animal Ethical Committee of the University Utrecht, The Netherlands (protocol number 2007.II.06.152/Vervolg1).

### 2.2 Searches in genomes and EST databases

BLAST searches were performed in selected genomes (www.ensembl.org) and EST databases (www.ncbi.nlm.nih.gov/BLAST) with the nucleotide sequence of the α1 and the α2 domains of human CD1D (**NM_001766**) and with the human TRAV10 segment (also called Vα24) used by NKT cells (**AE000659**). The results of the CD1D searches were included in a phylogenetic tree together with the α1 and the α2 domains of the known CD1 isoforms to assess with which CD1 isoforms they group. Also, the obtained potential CD1D genes were checked for the presence of a leader peptide, α1, α2, and α3 domain, and a transmembrane region. The nucleotide sequence of the α1 domain of the published sheep CD1d cDNA (**AJ006722**) was used to perform a BLAST search in the NCBI sheep EST database (www.ncbi.nlm.nih.gov/BLAST). The predicted amino acid sequences of the hits obtained from BLAST searches with the human TRAV10 segment in the genomes of selected species were all aligned and evaluated as described in Section 3.

### 2.3 Primer design, PCR and sequencing

Universal ruminant CD1D primers (Invitrogen) were designed based on the sequences of bovine pseudogene CD1D1 (accession number **DQ192544**). Forward primers “K” (5′-GGCGGAGATTCGAGGTCCG-3′) and “S” (5′-CGTTGGGGGAGGACGGAGG-3′) ended approximately 20 and 50 base pairs, respectively upstream of the start codons. The reverse primers “N” (5′-CTTCTCCAGCTCMGACTTCCC-3′) and “M” (5′-CAGGCCCAGGACTGGGGCCACTGG-3′) were designed to anneal to the end of the α2 domain and the beginning of the α3 domain, respectively. Primers for the full length CD1d transcripts of guinea pig, African elephant, rabbit, and horse were designed based on the predicted sequences in their genomes: CporCD1dFor#1 (5′-TGCTCAGAAGTCGCGGTCCC-3′), CporCD1dRev#2 (5′-AAATCTTAGCTACTCACAGGATATCTTGATAG-3′), LafrCD1dFor#1 (5′-GAGGCCAGGGTAGGACTTCAG-3′), LafrCD1dRev#1 (5′-CTTGGGCACTTGACGTCCTGAG-3′), OcunCD1dFor#2 (5′-GGGAGTGCAGCGCTAGTTTG-3′), OcunCD1dRev#1 (5′-TCACAAGATGCCTTGGTAGGAGC-3′), EcabCD1dFor#1 (5′-CTCCACACGCAGCGACATGAG-3′), EcabCD1dRev#1 (5′-TTGAGGTCGTGAGTTCCAGACAG-3′). TCR α chains were amplified using a V segment-specific forward primer and a reverse primer in the constant segment: CporTCRaVFor#1 (5′-CTTCTGAATAGGTGTGAATGGCAACC-3′), CporTCRaCRev#1 (5′-CTGACAGATTGACGTTAGAATCAAAATCG-3′), LafrTCRaVFor#1 (5′-CTTTCTGAATAGGGGTGAATGGCAAC-3′), LafrTCRaCRev#1 (5′-GTGACACGTTGGCTTTAGAATCGAAATC-3′), FcatTCRaVFor#1 (5′-GCAAAAACCAAGTGGAACAGCACC-3′), FcatTCRaCRev#1 (5′-CACTGTCAGGTTGTCGAAATTTAGGTTC-3′), EcabTCRaVFor#1 (5′-GAGGGAGAGAACTGCACGTTTC-3′), EcabTCRaCRev#1 (5′-TGCGGAACCCAATCAGTGACA G-3′), SscrTCRaVFor#1 (5′-GGTGAATGGCAAAAACCAAGTGGAAC-3′), SscrTCRaCRev#1 (5′-CT GAC AGG TTT TGG AGG TTG AGG TTC-3′), OariTCRaVregFor#1 (5′-CAATTATACAGTGAGCCCCTTTAACAAC-3′), OariTCRaCRev#1 (5′-GAACACGGTCACTGACAGGTTTTG-3′), BtauTCRaFor (5′-CAAATGGACGGTACACAGCGAC-3′), BtauTCRaCRev “J” (5′-TTTCAAAGCTTTTCTCTACCAGCTTGG-3′). PCR was performed with PFU Turbo polymerase (Stratagene) according to the protocol of the manufacturer using 20 ng genomic DNA or 10 ng cDNA in a reaction volume of 20 μl under the following cycling conditions: an initial denaturation of 7 min at 95 °C, followed by 40 cycles of 30 s at 95 °C, 45 s at different annealing temperatures (60 °C, 57 °C, 54.5 °C), 1 min at 72 °C, followed by a final elongation step of 7 min at 72 °C. PCR products were cut from a 1.5% agarose gel, purified with Gel DNA recovery kit (Zymo), and ligated in a Topo4blunt vector which was used to transform one shot Top10 cells (Invitrogen). DNA inserts of single colonies was sequenced by Baseclear (Leiden, The Netherlands).

### 2.4 Sequence analysis and homology modeling

Homology models of pig (*Sus scrofa*) and horse (*Equus caballus*) CD1d, as well their α chain of the invariant NKT cell TCR were modeled using the Swiss Model Server (Schwede et al., 2003), using both human CD1d and Vα24 TCR crystal structures as templates. The obtained CD1d and TCR models were superimposed onto their corresponding human counterparts in the CD1d/α-GalCer/Vα24 TCR crystal structure (PDB code **2PO6**) (Borg et al., 2007). No reorientation of the TCR was necessary to accommodate the TCR CDR loops, due to their similar orientation in both models. The CD1d surface residues in all three CD1d orthologs are mostly conserved, except for a glycine residue instead of the human tryptophan (W153), which is responsible for tilting the galactose of α-GalCer when bound to human CD1d (Koch et al., 2005) in comparison to mouse CD1d. Therefore, we manually modeled this galactose in the orientation that it adopts when bound to mouse CD1d, as mCD1d also has this conserved glycine residue (Zajonc et al., 2005). The models were visualized using PyMol (pymol.sourceforge.net).

The Translate Nucleic Acid Sequence Tool was used (http://biotools.umassmed.edu) for translation into amino acids. Alignments were performed and trees generated with ClustalW and Phylip. SignalP, available at http://www.cbs.dtu.dk/services/SignalP/ was used to predict leading fragments and cleavage sites.

## 3 Results

### 3.1 Invariant α chain analysis

The human TRAV10 V segment (also called Vα24) that is used by the human NKT invariant TCR was used to identify TCR α chain V segments in the genomes of cat, dog, horse, pig, cattle, guinea pig, African elephant, rabbit, and sheep. All resulting V α segment sequences were translated and aligned with the human TRAV10 segment. We considered all V segments with higher sequence homology to TRAV10 than to any other human V segment as candidate V segments for the NKT invariant chain in other species. Because the CDR1 region is encoded by the V segment and known to interact with α-GalCer (Borg et al., 2007; Kjer-Nielsen et al., 2006; Scott-Browne et al., 2007), we only included V segments in which at least two residues, including the P that was indicated as crucial in all studies, were identical to the human TRAV10 CDR1 region (VSPFSN). According to these criteria we identified one candidate V segment in cat, dog, horse, pig, guinea pig, African elephant, rabbit, and sheep, and three in cattle (Table 1a).

**Fig. 1:**
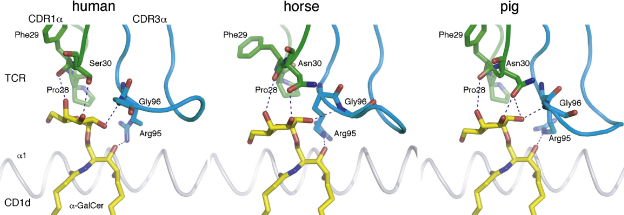
NKT cell receptor α chain binding to CD1d-bound α-GalCer. Residues of CDR1α (green) and CDR3α (cyan) that directly interact through hydrogen-bonding with α-GalCer, are represented as stick, colored by atoms (oxygen in red, nitrogen in blue). The α-GalCer ligand is shown as yellow sticks, while the CD1d α1-helix is shown in grey. The α2-helix of CD1d was removed for clarity. Hydrogen bonds are depicted as blue dashed lines. Only one residue in the porcine and equine CDR1α sequence (Asn30) differs from the human counterpart (Ser30) but the model suggests that it can still hydrogen bond with the α-GalCer ligand. Several other TCR residues that are involved in binding to CD1d residues are also conserved or similar but not shown. See sequence alignment of CD1d (Fig. 3) and NKT TCR (Table 1) for detailed sequence conservation.

Using a forward primer before or at the CDR1 region of the candidate V segments and a reverse primer in the constant segment, we amplified partial TCR α chains covering the CDR1, CDR2, CDR3, and part of the constant domain. For this purpose, PBMC-derived cDNA was available from cat, dog, horse, pig, guinea pig, African elephant, rabbit, sheep and cattle. CDR3 sequences that were highly homologous to the human and murine NKT CDR3α were obtained from horse (two out of four sequences) and pig (one out of 11 sequences). Six out of eight sequences obtained from cat had a two amino acid deletion in the CDR3 compared to the human and murine sequences. From cattle, one out of 15 sequences showed high homology to the human CDR3, but it had one extra amino acid. None of 15 sheep sequences, eight guinea pig sequences, eight rabbit sequences, and one African elephant sequence showed homology to the human invariant CDR3α (Table 1b). We were not able to derive TCR α chain sequences from dog. To predict whether the obtained CDR1α and CDR3α loops would be able to interact with a CD1d/α-GalCer complex, we generated models using the Swiss Model server (Schwede et al., 2003), and compared these to the available human data (Borg et al., 2007). The horse and pig invariant TCR α chain/CD1d/α-GalCer models suggest that these α chain sequences are fully functional invariant NKT cell TCR sequences, capable of binding α-GalCer, when presented by its species-matched CD1d molecule (Fig. 1). Even though otherwise highly conserved, the differences in CDR3α length of the obtained bovine and feline sequences make it difficult to predict whether the residues that normally interact with α-GalCer do so in these species, and therefore we cannot conclude that these sequences represent the bovine or feline NKT invariant chain.

### 3.2 CD1D pseudogenes in ruminants

**Fig. 2:**
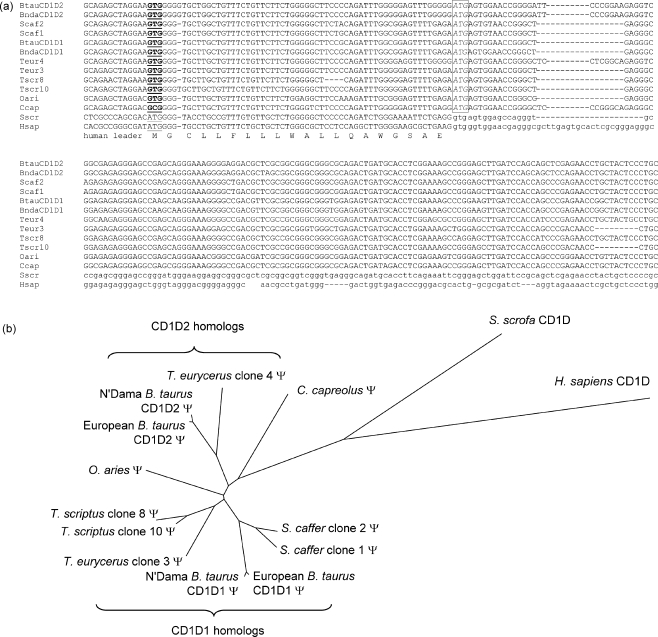
Ruminant CD1D pseudogenes (a) Alignment of the 5′ end of newly derived ruminant genomic CD1D sequences (full length sequences available under accession numbers **EU247610**–**EU247617** and **FJ028651**–**FJ028652**) with the previously published sequences of bovine CD1D1 (**DQ192544**), bovine CD1D2 (Van Rhijn et al., 2006), porcine CD1D (**AB221037**), and human CD1D (**NM_001766.3**). Functional human and porcine start codons: underlined; the mutated ruminant equivalent codons: underlined, bold; possible alternative start codon formed by the mutated donor splice site in ruminants: box; donor and acceptor splice sites in the human and porcine sequence: gray shade. The intron of the human and porcine CD1D sequence is shown in lower case and the translation of the human exons is shown on the lowest line. Btau: *Bos taurus*; Bnda: N’Dama breed of *B. taurus*; Scaf: *Syncerus caffer*; Teur: *Tragelaphus eurycerus*; Tscr: *Tragelaphus scriptus*; Oari: *Ovis aries*; Ccap: *Capreolus capreolus*; Sscr: *Sus scrofa*; Hsap: *Homo sapiens*. (b) Neighbor joining unrooted tree of porcine CD1D, human CD1D, and the newly derived ruminant genomic CD1D genes. Ψ: pseudogene.

PCR products were generated using genomic DNA from N’Dama cattle (*Bos taurus*), African buffalo (*Syncerus caffer*), sheep (*Ovis aries*), roe deer (*Capreolus capreolus*), bushbuck (*Tragelaphus scriptus*), and bongo (*Tragelaphus eurycerus*), using heterologous CD1D primers. Subsequent cloning of PCR products and sequencing of at least four independent bacterial colonies of each species resulted in CD1D sequences available at Genbank with accession numbers **EU247610**–**EU247617** and **FJ028651**–**FJ028652**. In case of small differences between sequences derived from one species, the sequence that was closest to the consensus sequence was submitted to Genbank. Alignment of the newly derived ruminant sequences with previously published CD1D sequences of humans and cattle (Fig. 2a) revealed that all newly derived ruminant CD1D sequences have the same disrupting mutations as the bovine CD1D genes. The start codon is mutated and the donor splice site of the first intron (the intron after the leading fragment) is mutated, rendering it an unspliceable intron. Interestingly, the mutated donor splice site of that intron forms ATG in all ruminant CD1D genes, and might function as an alternative start codon. This ATG is in the right reading frame and does in most cases not lead to any premature stop codons. However, the protein that would be synthesized is not predicted to contain a leading fragment by the SignalP program and can thus not be expressed at the cell surface (Signal peptide probability: 0.001, Signal anchor probability: 0.000). In N’Dama cattle and bongo we found one gene homologous to bovine CD1D1 and another gene homologous to bovine CD1D2. The obtained African buffalo and bushbuck sequences are homologous to bovine CD1D1. The roe deer and sheep sequences could not be classified as CD1D1 or CD1D2 (Fig. 2b).

The published sheep CD1d mRNA sequence with accession number **AJ006722** (Rhind et al., 1999) does not show disruptive mutations, while the sheep CD1D pseudogene we describe here does. Comparison of exons 1–3 of these two sequences, revealed that they were >98% identical at nucleotide level, suggesting that **AJ006722** may be a transcript of the gene we report here. To obtain additional data on the status of the sheep CD1D gene, we investigated CD1D transcripts in the sheep EST database. A BLAST search with the nucleotide sequence of exon 2, encoding the α1 domain of the **AJ006722** sequence resulted in five hits that were >98% identical at nucleotide level, suggesting that they were transcripts of the same gene. Three of these hits (**EE803429**, **DY491833**, and **DY491595**) contained a mutated start codon and an unspliceable intron between the leading fragment and the α1 domain. The other two hits did not contain any sequence upstream of the α1 domain. From this we conclude that in the EST database there are no functional CD1D transcripts corresponding to the **AJ006722** sequence, but there are transcripts of the pseudogene we describe in this paper. The only sheep CD1 proteins that have been demonstrated at protein level were CD1b and CD1e, isolated by immunoprecipitation with an antibody that recognizes multiple ruminant CD1 molecules (Rhind et al., 1999).

### 3.3 CD1D genes and CD1d transcripts in non-ruminant species

**Fig. 3:**
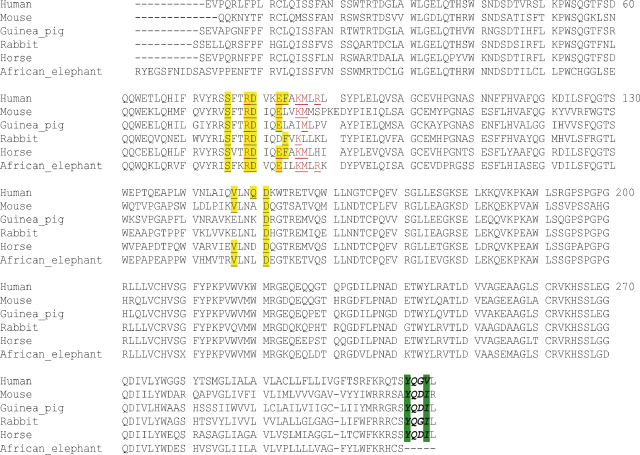
Comparison of CD1d sequences. The human and murine CD1d sequences were aligned with the newly derived guinea pig, rabbit, horse, and African elephant sequences (accession numbers **FJ028653**–**FJ028656**). Residues that are in the human CD1d sequence known to interact with the human NKT TCR CDR3α are in yellow/underlined, and with the human CDR2β in red/underlined (Borg et al., 2007). The YXXZ motif in the tail sequence is shown in green/bold/italics.

CD1D sequences were identified in the genomes of dog, cat, pig, guinea pig, horse, African elephant, rabbit, nine-banded armadillo, small Madagascar hedgehog, European shrew, and northern tree shrew (Table 2). A full length CD1D sequence without any of the characteristics of pseudogenes could be found in pig, horse, and nine-banded armadillo. The CD1D sequences of the other mammals were incomplete because of gaps in the genomic sequences. However, the available parts of the sequences did not show any of the characteristics of pseudogenes. In order to obtain the full length coding sequence of the incomplete genes, and proof that the CD1D genes are transcribed and properly spliced in vivo, we successfully cloned full-length CD1d transcripts from guinea pig, rabbit, horse, and African elephant PBMC (accession numbers **FJ028653**–**FJ028656**). Alignment of these sequences with the human and murine CD1d sequences (Fig. 3) shows that the residues on the surface of CD1d that interact with the NKT TCR are highly conserved. Contrary to all other CD1d sequences the African elephant CD1d sequence has a truncated cytoplasmic tail and lacks a YXXZ motif. The YXXZ motif in the tail sequence of murine and human CD1d is needed for interaction with AP-2 and thus trafficking to the late endosome (Chiu et al., 1999; Rodionov et al., 1999).

## 4 Discussion

In this paper we show that the NKT/CD1d system is present in horse and pig. Equine and porcine NKT invariant α chains and CD1d transcripts are sequenced and their models suggest that they are likely to function as their human and murine counterparts. In addition, we sequenced full length CD1d cDNA of African elephant, guinea pig, and rabbit, and we show that in the genomes of dog, cat, African elephant, nine-banded armadillo, Madagascar hedgehog, European shrew, and Northern tree shrew (partial) functional CD1D genes are present, suggesting that these species may also have a functional NKT/CD1d system. However, in the six ruminant species we studied here, all CD1D genes we identified were non-functional, which strongly suggests that ruminants may not have NKT cells.

Human and murine CD1d-restricted NKT cells can be detected using α-GalCer-loaded CD1d tetramers. Even though human and murine CD1d tetramers cross-react between these two species, lack of detection of NKT cells in ruminants using murine or human CD1d tetramers is not conclusive because these reagents may not cross-react with ruminants. Using the same molecular approach that led to the identification of the invariant NKT α chain in pig and horse we were unable to identify invariant NKT α chain homologs in guinea pig, cat, rabbit, African elephant, cattle and sheep. So, even though these species do express V segments homologous to TRAV10 (Vα24), we have not found these V segments in combination with the canonical NKT CDR3α. It is possible that we did not obtain invariant NKT α chain sequences because the TRAV10-homologous V segment is used often by other, non-NKT cells in these species, our sample size is not big enough, and/or the NKT cells are under represented in PBMC. Therefore, based on TCR α chain sequences only, we cannot conclude that NKT cells are absent in these species. However, the combination of the fact that ruminants lack functional CD1D genes, and the observed absence of an invariant α chain sequence among 26 different ruminant (bovine and ovine) TRAV10 homolog-containing TCR α chain sequences points strongly to absence of NKT cells in ruminants.

CD1d presents lipids with an α-glycosidic linkage to NKT cells and may therefore be an important molecule to stimulate the immune system in response to α-proteobacteria that contain these compounds. Ruminants are very sensitive to infection with these pathogens. Previously we have shown that European cattle lack functional genes for CD1D. Because we found CD1D pseudogenes and no functional CD1D genes in African N’Dama cattle (*Bos taurus*), two other species of the family *Bovinae* (bongo, bushbuck, and African buffalo), a member of the superfamily *Bovidae* that does not belong to the family of *Bovinae* (sheep), and a ruminant that is a member of the superfamily *Cervidae* (roe deer), we conclude that CD1d proteins are probably absent in all ruminants, though we are aware that we have not formally proven this. In the absence of a fully finished and assembled genome, it is difficult to prove that a functional CD1D gene is absent in a certain species. Southern blotting detects hybridizing sequences, but does not discriminate between functional genes and pseudogenes and between comigrating restriction fragments. Especially the latter poses problems because homology of CD1 genes can be exceptionally high, and these genes will be cut in an identical way by the restriction enzymes used, leading to an underestimation of the real number of CD1 genes. This probably explains that the guinea pig was previously suspected of having no CD1D gene (Dascher et al., 1999) based on Southern blot data, while we report a guinea pig CD1D gene and transcript here.

Our data on the presence of a CD1D pseudogene in sheep, carrying a mutated start codon and an unspliceable intron, seem to be in contradiction with published data on sheep CD1d. A full-length cDNA sequence of sheep CD1d has been published and is predicted to translate into a normal CD1d protein (Rhind et al., 1999). However, this cDNA sequence has been assembled in silico from partial PCR products. The full-length cDNA sequence in which the first intron was properly spliced out has never been obtained (Rhind, personal communication). In the sheep EST database we could only find CD1D pseudogene transcripts, and no functional transcript analogous to the published one. Together, this suggests that the published sheep CD1d cDNA may derive from transcripts of a sheep CD1D pseudogene, and are consistent with the possibility that sheep do not have functional CD1d.

The artiodactyl pig (*S. scrofa*) is the closest relative of the ruminants that we studied, and it has a functional gene and no CD1D pseudogene. This dates the loss of a functional CD1D gene by point mutations, and thus the emergence of a CD1D pseudogene approximately 65 million years ago, when the ancestors of *Suidae* and *Ruminantia* diverged (Kumar and Hedges, 1998). This is consistent with the fact that we have only found CD1D pseudogenes in all ruminants studied and argues against the presence of a functional sheep CD1D gene.

To emphasize the special status of group 2 CD1 molecules as compared to group 1 CD1 molecules, it has often been stated that CD1d molecules and NKT cells are universally present in mammals, while this is not the case for group 1 CD1 molecules. Lack of functional CD1D genes in a considerable group of animals as we show here would suggest that there is no reason for a special status for CD1d proteins based on universal distribution among mammals. In addition to different expression patterns and being slightly separated based on sequence homology, group 2 CD1 molecules (CD1d) are thought to differ fundamentally from group 1 CD1 molecules (CD1a, CD1b, and CD1c) in that they stimulate an invariant T cell population. However, in addition to being able to activate NKT cells with an invariant TCR, it has been shown that CD1d can also stimulate other, non-invariant T cells (Baron et al., 2002; Huber et al., 2003; Jahng et al., 2004; Van Rhijn et al., 2004). Whether CD1d is the only member of the CD1 family of proteins that can stimulate an invariant T cell population, remains open: it is possible that in the future invariant group 1 CD1-restricted T cell populations will be discovered, and if so, this would question whether group 1 and group 2 CD1 proteins really perform fundamentally different functions.

## Figures and Tables

**Table 1:**
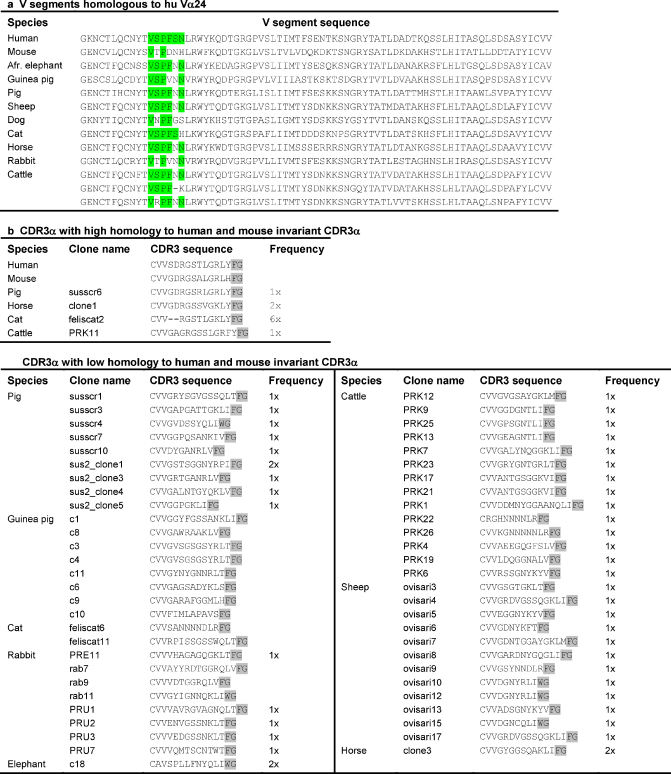
Sequences of V segments homologous to TRAV10 and CDR3 of T cells using these V segments.

a Amino acid sequences of TRAV10-homologous V segments in several species, identified by searching the available genomic data. In green: CDR1. b TCR α chain sequences using the TRAV10 homologs were derived from PBMC from multiple species. The CDR3 of these TCR α chains that are highly homologous to the CDR3 of the human and mouse NKT TCR α chain are aligned (top panel). The human and mouse sequences that are included were derived from literature. CDR3 that were not homologous to the CDR3 of the human and mouse NKT TCR α chain, but were used by TRA10 homologous V segments are shown for comparison (lower panel). Green: CDR1; Grey: the first two amino acids of the FGXG motif, forming the end of the CDR3.

**Table 2:** Newly identified CD1D sequences in mammalian genomes.

Species	NCBI accession or ensemble gene ID	Link	Functionality
Dog (Canis familiaris)	AC183576	http://www.ncbi.nlm.nih.gov/sites/Nucleotide	Incomplete^a^
Cat (Felis catus)	ENSFCAG00000012532	http://www.ensembl.org/Felis_catus	Incomplete^a^
Pig (Sus scrofa)	NM_001102680.1	http://www.ncbi.nlm.nih.gov/sites/Nucleotide	Functional^b^
Guinea pig (Cavia porcellus)	ENSCPOG00000013837	http://www.ensembl.org/Cavia_porcellus	Functional^a^^,^^b^
Horse (Equus caballus)	NW_001800080.1	http://pre.ensembl.org/Equus_caballus	Functional^b^
African elephant (Loxodonta africana)	ENSLAFG00000013716	http://www.ensembl.org/Loxodonta_africana	Functional^a^^,^^b^
Nine-banded armadillo (Dasypus novemcinctus)	ENSDNOG00000018694	http://www.ensembl.org/Dasypus_novemcinctus	Functional^c^
Madagascar hedgehog (Echinops telfairi)	ENSETEG00000015412	http://www.ensembl.org/Echinops_telfairi	Incomplete^a^
European shrew (Sorex araneus)	ENSSARG00000000647	http://www.ensembl.org/Sorex_araneus	Incomplete^a^
Northern tree shrew (*Tupaia belangeri*)	ENSTBEG00000001753	http://www.ensembl.org/Tupaia_belangeri	Incomplete^a^

a The genomic sequence contained gaps. The available part does not contain any of the characteristics of a pseudogene.

b Full-length transcripts of this gene that are predicted to translate into a functional protein have been described in this paper.

c The gene is complete and did not contain any of the characteristics of a pseudogene, but it is unknown whether the gene is transcribed and translated in vivo.
